# The transcriptional activator ZNF143 is essential for normal development in zebrafish

**DOI:** 10.1186/1471-2199-13-3

**Published:** 2012-01-23

**Authors:** Kari M Halbig, Arne C Lekven, Gary R Kunkel

**Affiliations:** 1 Department of Biochemistry and Biophysics, Texas A&M University, College Station, TX 77843-2128, USA; 2 Department of Biology, Texas A&M University, College Station, TX 77843-3258, USA

## Abstract

**Background:**

ZNF143 is a sequence-specific DNA-binding protein that stimulates transcription of both small RNA genes by RNA polymerase II or III, or protein-coding genes by RNA polymerase II, using separable activating domains. We describe phenotypic effects following knockdown of this protein in developing *Danio rerio *(zebrafish) embryos by injection of morpholino antisense oligonucleotides that target znf143 mRNA.

**Results:**

The loss of function phenotype is pleiotropic and includes a broad array of abnormalities including defects in heart, blood, ear and midbrain hindbrain boundary. Defects are rescued by coinjection of synthetic mRNA encoding full-length ZNF143 protein, but not by protein lacking the amino-terminal activation domains. Accordingly, expression of several marker genes is affected following knockdown, including GATA-binding protein 1 (*gata1*), cardiac myosin light chain 2 (*cmlc2*) and paired box gene 2a (*pax2a*). The zebrafish *pax2a *gene proximal promoter contains two binding sites for ZNF143, and reporter gene transcription driven by this promoter in transfected cells is activated by this protein.

**Conclusions:**

Normal development of zebrafish embryos requires ZNF143. Furthermore, the *pax2a *gene is probably one example of many protein-coding gene targets of ZNF143 during zebrafish development.

## Background

The vertebrate transcriptional activator protein, ZNF143 (also known as STAF for selenocysteine tRNA gene transcription activating factor, or SBF for SPH-binding factor) operates at a multitude of small RNA and protein-coding gene promoters [1-5]. Two separable activation domains within this protein stimulate transcription selectively at either small RNA or mRNA promoters [6]. Initially, attention was focused on the function of ZNF143 for small RNA gene transcription, especially for vertebrate snRNA and selenocysteine tRNA genes [7-9]. Then, several mRNA genes were identified whose proximal promoters contained SPH sites [10-15]. Possibly because of the highly degenerate and relatively long DNA-binding site recognized by ZNF143, it was not recognized for many years that approximately 2000 mammalian protein-coding genes contain SPH (*Sph*I Postoctamer Homology [16]) elements, or STAF Binding Sites (SBS), in their promoters [5].

Little is known concerning the phenotypic role(s) of ZNF143 during cellular growth and animal development. A number of cell-cycle-associated gene promoters are regulated by ZNF143 [17-19]. Furthermore, ZNF143 is an important regulator of mammalian embryonic stem cell renewal [20,21]. At the molecular level, recent work has demonstrated that this activator protein interacts with the chromodomain-helicase-DNA binding protein 8 (CHD8), and implicates that human *U6 *gene transcription is stimulated by ZNF143 through this interaction [22]. Many potential small RNA and protein-coding gene promoters are targeted, but which are most pivotal *in vivo*?

We used zebrafish embryos as a model system to investigate the role of ZNF143 during vertebrate development. Injection of translation-blocking morpholino oligonucleotides (MOs) resulted in a pleiotropic phenotype including axial defects as well as abnormalities in heart, blood, ear and midbrain hindbrain boundary (MHB). Coinjection of synthetic mRNA encoding zebrafish ZNF143 rescued MO-induced defects, and rescue was dependent on the amino-terminal region of the protein containing activation domains. Expression levels or patterns of the *gata1*, *cmlc2*, and *pax2a *genes were altered following MO knockdown of zebrafish ZNF143. The *pax2a *gene is likely to be a direct target for ZNF143 because this protein binds the promoter *in vitro *and specific mutations in SPH sites resulted in reduced transcription in transient transfection experiments.

## Results

### Identification of mRNA gene activation region in ZNF143

**Figure 1:**
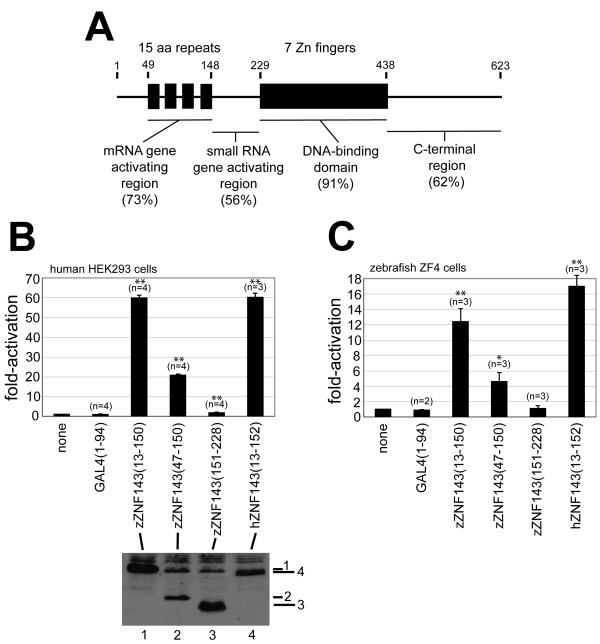
**Identification of transcriptional activating domain at the amino-terminus of zebrafish ZNF143**. (**A**) Primary structure of zebrafish ZNF143. Numbers on top depict the amino acids at the beginning and end of prominent primary structure features of the protein. Numbers in parentheses are percentages of identical amino acid residues between the zebrafish and human proteins for various regions of primary structure. (**B**) HEK293 cells were transfected with 200 ng of pGL3/G5AdMLT firefly luciferase reporter plasmid, 10 ng of the particular pCI/GAL4DBD/zZNF143 effector plasmid DNA noted in the figure, and 200 ng of pRL-SV40 renilla luciferase reporter. The fold-activation was determined by comparing the firefly luciferase/renilla luciferase ratios for each sample to that ratio for samples where no effector plasmid was added. Bar height shows the mean number from different transfected samples (number of experiments reported in parentheses), and the error bar represents the standard deviation from the mean. Double asterisks signify p-value <0.01, and single asterisks signify a p-value <0.05 relative to samples transfected with GAL4DBD only. The panel below shows the relative expression of each GAL4DBD/zZNF143 fusion protein in HEK293 cells by immunoblot analysis using anti-GAL4DBD antibodies. (**C**) Zebrafish ZF4 cells were transfected, and relative expression levels analyzed exactly as described for (**B**). We were unable to detect GAL4DBD/zZNF143 fusion proteins in transfected ZF4 cells, possibly because of the much lower transfection efficiency compared to HEK293 cells.

The zebrafish *znf143 *cDNA has been identified, and the predicted amino acid sequence contains a high degree of similarity with the human protein (71% overall identity by our measurement) [23]. Furthermore, the zebrafish protein contains highly conserved regions that correspond to the previously identified DNA-binding domain (DBD), mRNA gene activation domain (15 aa repeats) and small RNA gene activation domain of the Xenopus and human proteins [23] (Figure 1A). To verify the mRNA gene activating potential of zebrafish ZNF143 and demarcate boundaries of this region, we fused fragments encoding zebrafish ZNF143 to the *S. cerevisiae *GAL4p DNA binding domain (amino acids 1-94), and performed transient transfection assays with such expression plasmids and a firefly luciferase reporter gene transcribed from a minimal promoter driven by GAL4 binding sites. Because transcriptional activating domains of the Xenopus protein were localized to the amino-terminal end previously [6], we investigated this region only. Amino acids 13-150 of zebrafish ZNF143 contains a potent mRNA gene activation region that functions in both human embryonic kidney (HEK293) cells and zebrafish ZF4 cells (Figure 1B, 1C). The region including only the four 15 aa repeats (amino acids 47-150) was approximately three-fold less active in both cell types. However, it is possible that this difference could be due to a lower expression level of this fragment (Figure 1B). Importantly, the region of zebrafish ZNF143 between the 15 aa repeats and the zinc finger domain (amino acids 151-228) demonstrated minimal mRNA promoter activation. Within this region has been identified a small RNA gene activating domain in the Xenopus protein [6].

### Knockdown of ZNF143 in zebrafish embryos

To begin our studies with zebrafish embryos, we investigated the expression of *znf143 *RNA using whole-mount in situ hybridization. This RNA was expressed ubiquitously in the early stages that we examined, including 2-4 cell stage embryos and through gastrulation stages (results not shown). *znf143 *RNA must be inherited maternally, since it was detected prior to onset of zygotic transcription.

**Figure 2:**
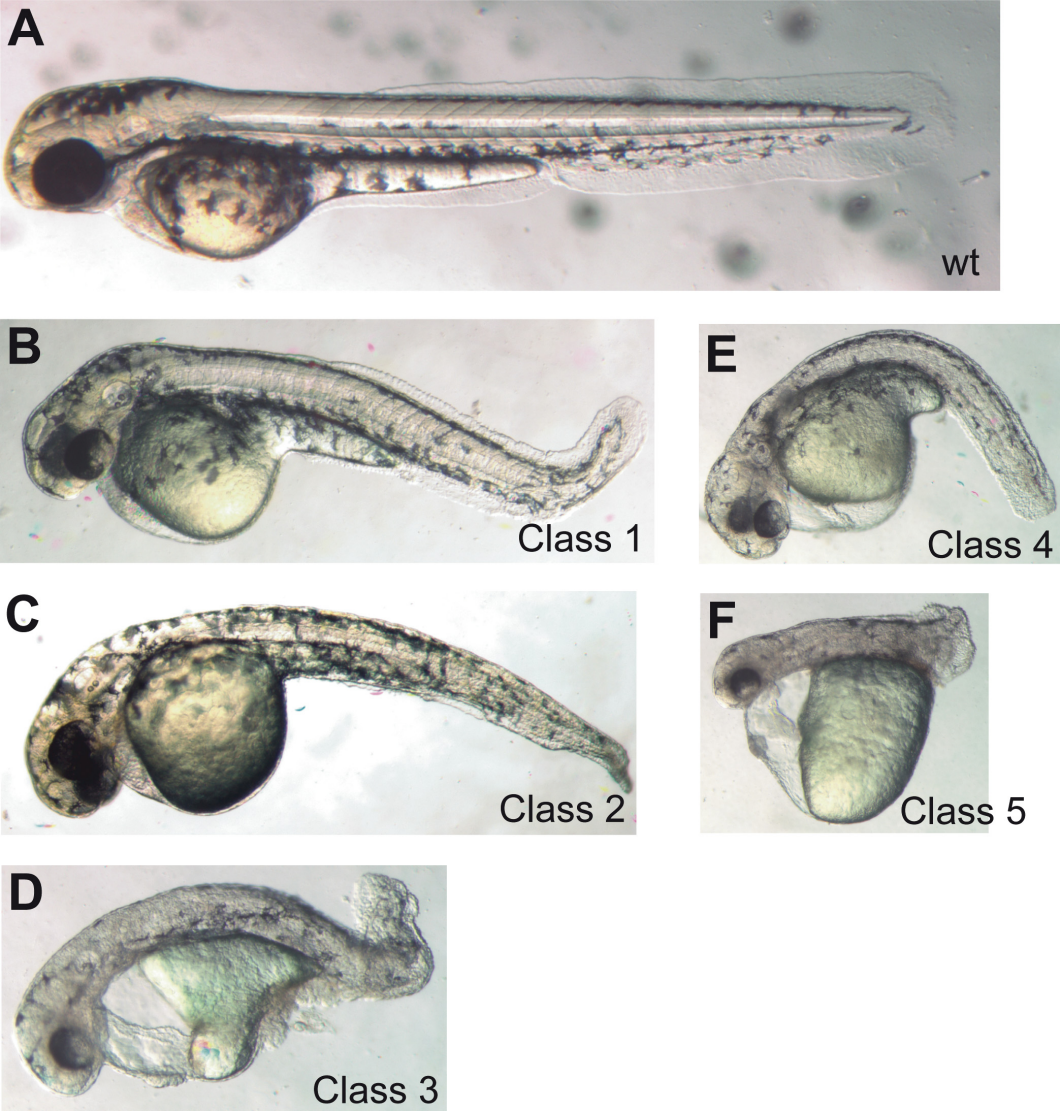
**MO-induced knockdown of ZNF143 produces multiple phenotypes in zebrafish embryos**. Zebrafish embryos were injected with a combination of "Start" and "5'UTR" morpholino oligonucleotides, each at 2 ng/nL. ZNF143 morphants at approximately 48 hpf are displayed according to increasing level of severity of axial development and other defects as described in the text (classified 1-5, respectively).

Next, we co-injected a pair of morpholino oligonucleotides (MOs) that targeted the translational start site and 5' untranslated region (5'UTR) of *znf143 *mRNA in order to reduce protein levels during embryonic development. Up to nearly 90% of injected embryos exhibited a range of defects at 48 hours post fertilization (hpf) (Figure 2). Microinjection of each individual MO resulted in the same range of phenotypes, but at a much lower efficiency (results not shown), supporting the assertion that the observed phenotypes are specific to ZNF143 knockdown. No mutant phenotypes were found when a control MO was injected at the same concentration (results not shown). For quantitation purposes, we sorted the morphant embryos into six classes that are primarily distinguished by increasing severity of axial and other defects. Class 1 embryos show a distinct kink in the tip of the tail (Figure 2B). Class 2 embryos have the same tail kink and also show disorganization in the somites (Figure 2C). Class 3 embryos have a greater degree of tissue disorganization as well as significant pericardial edema (Figure 2D). Class 4 embryos have the added phenotype of severe brain morphology defects, including absent or poorly formed midbrain-hindbrain boundary (MHB) (Figure 2E). Class 5 embryos display the additional phenotype of a considerably shortened axis (Figure 2F). Class 6 morphants (not shown) are morphological monsters, lacking both head and tail structures. In addition to the described effects, we also noted reduced blood formation, rate of circulatory flow and abnormal ear development in significant proportions of morphant embryos falling in the more severe classes (classes 3-6).

Early developmental stages, through most of epiboly, proceeded normally for the ZNF143 morphant embryos, compared to embryos injected with the control MO. However, we detected a delay of approximately 30 min to reach bud stage. Furthermore, subsequent stages for ZNF143 morphants at 6-somite, 10-somite, 18-somite, and 22-somite were delayed approximately one hour.

**Figure 3:**
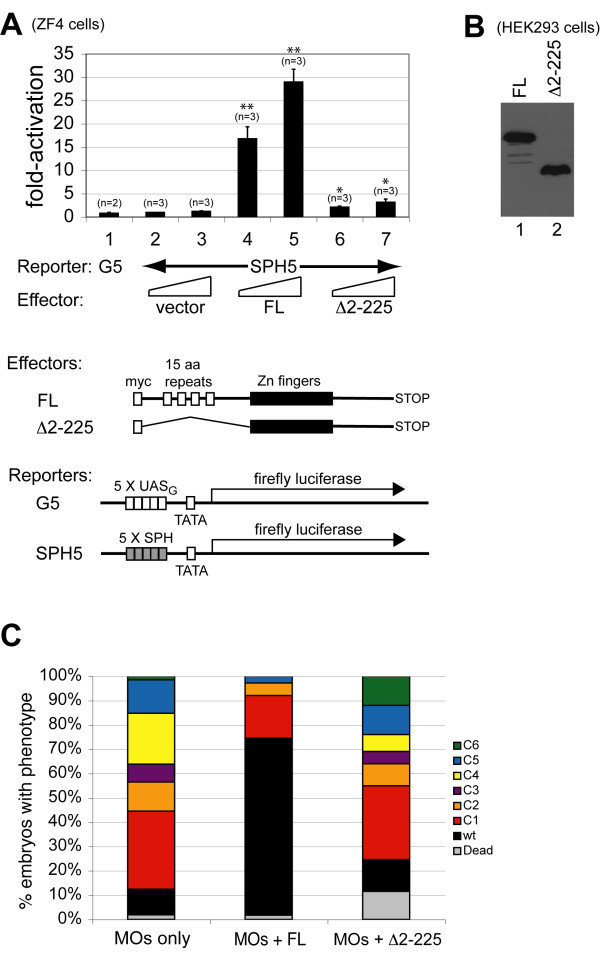
**Rescue of MO phenotypes by co-injection of synthetic zebrafish ZNF143 mRNA**. (**A**) Transcriptional activation of myc-tagged zebrafish ZNF143 deletion proteins in transfected ZF4 cells. Cells were transfected with 200 ng of firefly luciferase reporter plasmid, plus 200 ng of pRL-SV40 renilla luciferase reporter plasmid, plus various ZNF143 effector plasmids as noted in the figure at 5 ng (columns 2, 4, 6) or 25 ng (columns 3, 5, 7). Fold-activation was determined by comparing the firefly/renilla luciferase ratio for each sample to that ratio for the sample shown in column 2. Bar height shows the mean number from different experiments, and error bars report the standard deviation from the mean (when n = 3), or the range (when n = 2). Double asterisks signify a p-value <0.01, and single asterisks signify a p-value <0.05 relative to samples transfected with vector only (lane 2). (**B**) Relative expression of myc-tagged zebrafish ZNF143 proteins in transfected HEK293 cells. (**C**) Zebrafish embryos were injected with a combination of "Start" and "5'UTR" MOs along with either no RNA (MOs only), synthetic mRNA encoding wt zebrafish ZNF143 (MOs + FL), or synthetic mRNA encoding zebrafish ZNF143/Δ2-225 (MOs + Δ2-225). Embryos were scored as either dead, wt, or classes 1-6 (see Figure 2 for examples of these classifications) at 48 hpf. Results were averaged from three separate injection experiments for each condition (approximately 100 embryos per injection). The ranges in % wt phenotype for each condition in various experiments were: MOs only: 9-11%; MOs + FL: 69-72%; MOs + Δ2-225: 11-15%.

In order to verify the specificity of MO-knockdown, we co-injected synthetic mRNA encoding zebrafish ZNF143 with the MOs to determine whether protein synthesized from the exogenously supplied mRNA could rescue the phenotypes. We used mRNAs encoding either full length ZNF143 or a truncated variant lacking the transcription activation domains (Δ2-225) in our rescue assays. Both mRNAs lack the normal 5'UTR sequence and encode a myc tag at the amino-terminus of the protein, such that the morpholinos will not block their expression. We first confirmed that the full-length myc-tagged protein is functional for transcriptional activation in transfected ZF4 cells (Figure 3A, compare lanes 2 and 3 with lanes 4 and 5). In addition, the Δ2-225 protein was defective for transcriptional activation in transfected ZF4 cells (Figure 3A, lanes 6 and 7). We detected nearly equivalent levels of expression of the myc-tagged full-length and Δ2-225 proteins in transfected HEK293 cells (Figure 3B). Thus, our constructs should be expressed at equivalent levels when expressed in embryos, even though for unknown reasons we have been unable to detect these proteins by their myc-tags in injected embryos and in transfected ZF4 cells. Importantly, when co-injected with the MOs, full-length zebrafish ZNF143 rescued the general morphant phenotypes in approximately 70% of the injected embryos, and the remaining defects were primarily the least severe classes (Figure 3C, compare columns 1 and 2). The rescue was dependent on the presence of the amino-terminal region of zebrafish ZNF143 containing both activation domains, since co-injection of mRNA lacking codons 2-225 did not rescue the morphant phenotypes (Figure 3C, column 3). These results show that the amino-terminal myc tag does not inactivate the function(s) of zebrafish ZNF143 *in vivo*, and the observed MO-induced phenotypes are specific to the loss of ZNF143 function and more specifically to ZNF143 transcriptional regulatory capability.

### *pax2a *expression is compromised after ZNF143 knockdown

**Figure 4:**
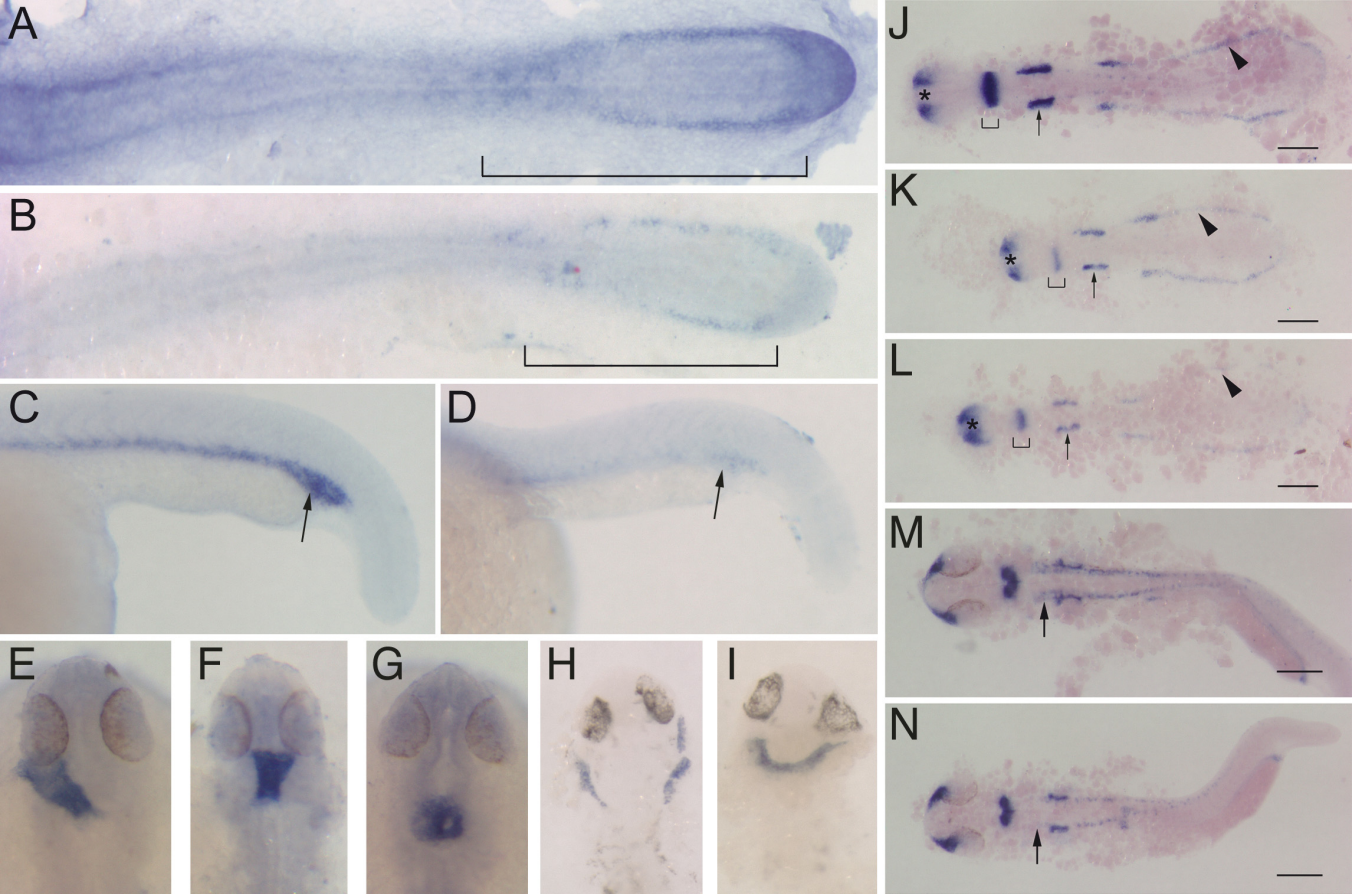
**Expression analysis of ZNF143 requirement during embryonic development**. In situ hybridizations to detect *gata1 *(**A-D)**, *cmlc2 *(**E-I**), and *pax2a *(**J-N**) expression. (**A, C, E, J, M**) Wild-type. (**B, D, F, G, H, I, K, L, N**) ZNF143 morphants. (**A, B**) 16 somite stage, flat mounted embryos, dorsal view, anterior left. Brackets indicate gata1^+ ^hematopoietic progenitors. Note reduced expression in the morphant (**B**). (**C, D**) 24 hpf, lateral views of tails. Note reduced *gata1 *expression in the ICM (arrows) of ZNF143 morphants (**D**) compared to wt (**C**). (**E-G**) approximately 27 hpf, dorsal views, anterior up. Note heart laterality in wt (**E**). (**F, G**) Two different phenotypes observed in ZNF143 morphants. While they show differences in tube morphogenesis, both lack laterality. (**H, I**) approximately 37 hpf, dorsal views, anterior up. Two different phenotypes in ZNF143 morphants. (**J-L**) 16 somite stage, flat mount, dorsal view, anterior left. Compared to the wt (**J**), ZNF143 morphants (**K, L**) display reduced *pax2a *expression in the pronephros (arrowheads), otic vesicles (arrows) and MHB (brackets). Note that expression in these domains is reduced in the morphants even though expression in the optic stalks is similar to wt (asterisks). (**M, N**) Approximately 27 hpf, dorsal views, anterior left. Expression of *pax2a *appears largely normal in ZNF143 morphants (**N**), with the exception of a substantial decrease in expression in hindbrain neurons (arrows). Scale bars: 100 μm in **J-L**, 200 μm in **M, N**.

To determine whether the general morphological defects of ZNF143 morphants could be correlated with specific changes in gene expression, we performed whole mount in situ hybridization to detect developmental gene expression in morphant embryos at several stages. Using goosecoid (*gsc*) and orthodenticle homolog 2 (*otx2*) probes to monitor prechordal plate mesoderm and neuroectoderm [24,25], respectively, during gastrulation (shield stage and 75% epiboly for *gsc*, and 75% epiboly for *otx2*), we detected no differences between ZNF143 morphants and embryos injected with a control MO (results not shown). In addition, hindbrain development of rhombomeres 3 and 5 appeared normal during early somitogenesis (6- and 10-somite stages) using an early growth response 2a (*egr2a*; or *krox20*) probe [26], although a delay in staining of rhombomere 5 was apparent in the 6-somite embryos (results not shown). This minor variation is consistent with the overall delay detected. Furthermore, we examined the expression of *myoD*, a marker for somitic muscle [27]. At all stages examined, we detected no discernable difference between wt and ZNF143 morphant embryos (5-6 somite, n = 20; 16 somite, n = 13; 27 hpf, n = 20; data not shown). Next we analyzed the expression of *gata1*, a marker for hematopoietic progenitors [28], since blood development is compromised in ZNF143 morphants. At the 16-somite stage, *gata1 *labels populations of hematopoietic progenitors in the posterior mesoderm flanking the midline (Figure 4A). Consistent with our observation that blood specification is aberrant, ZNF143 morphants exhibited a significant reduction in *gata1 *expression at this stage (Figure 4B; 50% penetrance, n = 20). At 24 hpf, *gata1 *is expressed in cells of the intermediate cell mass (ICM; Figure 4C), but expression continued to be reduced significantly in ZNF143 morphants (Figure 4D; 37% penetrance, n = 19). Thus, the reduction in blood cells we observed in ZNF143 morphants correlates with a significant reduction in *gata1 *expression that labels hematopoietic progenitors.

We next analyzed expression of cardiac myosin light chain 2 (*cmlc2*), a marker for cardiomyocytes, since heart development appeared disrupted after reduction of ZNF143. At 24 hpf, the heart comprises a tube that loops toward the left side of the embryo (Figure 4E). Heart progenitors formed normally in ZNF143 depleted embryos, but morphogenesis of the heart was severely compromised in 55% of MO-injected embryos (Figure 4F, 4G; n = 38). Phenotypic defects varied in this group from a failure to migrate to the left side of the embryo (Figure 4F), to a failure in tube morphogenesis (Figure 4G). These phenotypic defects in the ZNF143 morphants are not a result of prolonged delay of heart development, because embryos probed for *cmlc2 *expression at 30 hpf and 37 hpf did not exhibit normal asymmetric looping (26/27 defective). At these later stages, 26% displayed cardia bifida (Figure 4H, 4I).

Because ZNF143 morphants displayed morphological abnormalities in the brain, including defects in the MHB, we analyzed the expression of *pax2a*, a critical regulator of MHB formation. At the 16-somite stage, *pax2a *expression marks the optic stalks, mid-hindbrain domain, otic vesicles, pronephros and a small number of spinal cord neurons (Figure 4J). After reduction of ZNF143 function, *pax2a *expression was reduced in most expression domains of the majority of embryos (68%, n = 19). Expression of *pax2a *in the MHB was reduced substantially in ZNF143 morphants, and to a lesser degree, expression in the otic vesicles, and pronephros (Figure 4K, 4L). In contrast, expression in the optic stalks did not appear to be significantly diminished compared to wt. Importantly, this phenotype was rescued by coinjection of wt ZNF143 mRNA (100% rescue, n = 14). At earlier stages (bud, 6-somite, 10-somite) *pax2a *expression was comparable between ZNF143 morphants and embryos injected with control MO. Furthermore, by 24 hpf, *pax2a *expression was not significantly different between wt and ZNF143 morphants, with the exception that *pax2a *expression in a group of hindbrain neurons was reduced or absent in the morphants (Figure 4M, 4N, arrows; 100% penetrance, n = 18). Thus, normal *pax2a *expression, particularly at the MHB, depends on ZNF143 function.

### The zebrafish *pax2a *gene promoter contains binding sites for ZNF143 and is controlled by this protein in transfected cells

**Figure 5:**
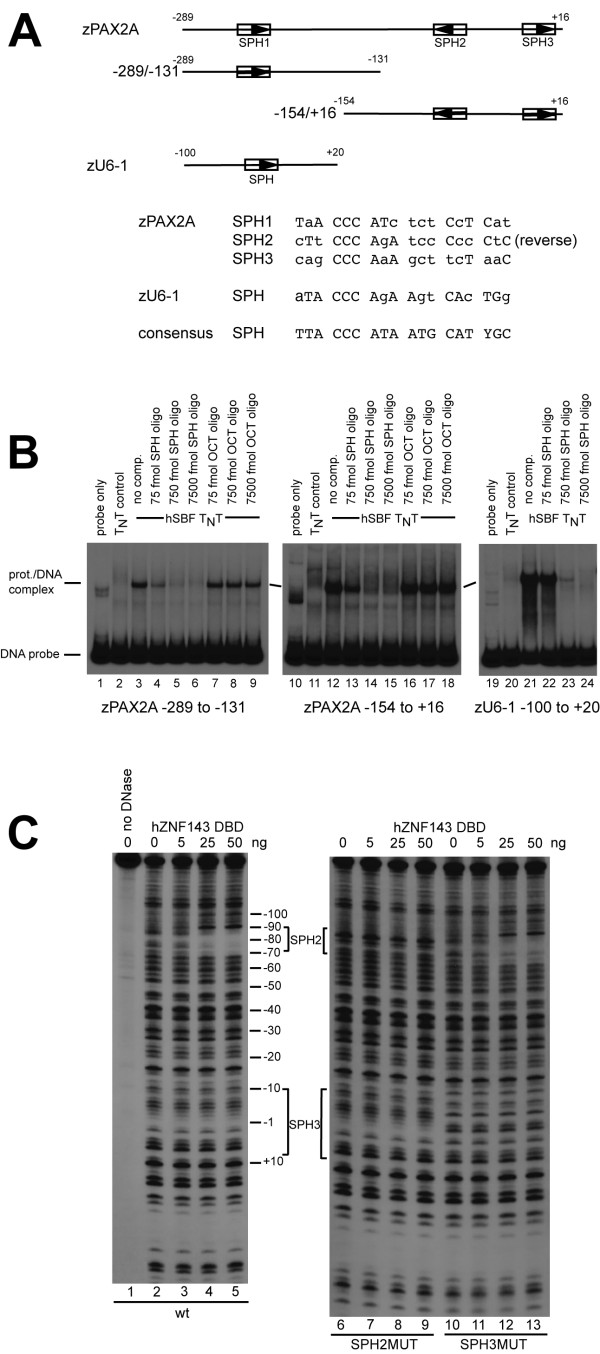
**ZNF143 binds to the zebrafish *pax2a *promoter *in vitro***. (**A**) Map of zebrafish *pax2a *proximal promoter showing three potential SPH sites. The sequences of the potential SPH sites are compared to the zebrafish *U6-1 *element [29] and the consensus SPH sequence from [37]. Capital letters depict nucleotides matching the consensus. To facilitate binding assays two separate radiolabeled zebrafish *pax2a *probes were prepared by PCR as diagrammed. (**B**) Electrophoretic mobility shift assay. Approximately 3 fmol of radiolabeled probes depicted in (**A**) were incubated with 3 μL of human ZNF143-(88-638) expressed by *in vitro *transcription/translation (lanes 3-9; 12-18; 21-24) or with unprogrammed extract (lanes 2, 11, 20), and electrophoresed. In addition, in samples shown in lanes 4-6, 13-15, and 22-24, an unlabeled double-stranded oligonucleotide with the sequence including the human *U6-1 *SPH element was added with the labeled probe in amounts noted, or similarly, an unlabeled oligonucleotide containing the unrelated, OCT sequence was added to samples in lanes 7-9 and 16-18. (**C**) DNase I footprinting assay. Approximately 3 fmol of singly end-labeled zebrafish *pax2a *probe from -154 to +16 were incubated with amounts of purified human ZNF143 DBD protein as indicated. The wt *pax2a *promoter was used for samples in lanes 1-5, whereas SPH2 or SPH3 mutant promoters were used for samples in lanes 6-9, or lanes 10-13, respectively.

Since *pax2a *gene expression was affected after ZNF143 knockdown, we investigated whether this transcription factor might control zebrafish *pax2a *transcription. Visual inspection of the proximal promoter region sequence indicated three possible SPH motifs (Figure 5A). Radiolabeled probes were prepared that contained either the SPH1 element alone (-289/-131 fragment), or both SPH2 and SPH3 sites (-154/+16 fragment). Both probes bound human ZNF143, showing a single complex that was competed with a specific human *U6-1 *SPH oligonucleotide, but not with an unrelated sequence containing the octamer (OCT) transcription control element (Figure 5B). Specific oligonucleotide competition for the *pax2a *DNA fragments was similar to that for the zebrafish *U6 *small nuclear RNA gene promoter used as a positive control for these experiments (Figure 5B, rightmost panel; [29]). Since only a single complex dependent on addition of a nonsaturating amount of ZNF143 was detected with the - 154/+16 fragment, we suspected that only one of the two potential SPH elements was bound. DNaseI footprinting was used to determine which SPH element in the - 154/+16 fragment was bound by the protein. Results from footprinting experiments demonstrated binding of the human ZNF143 DNA-binding domain to the SPH2 element, while the SPH3 site was unoccupied at concentrations used for the experiment (Figure 5C. left panel). Therefore, two sites (SPH1 (likely) and SPH2) within the zebrafish *pax2a *promoter are bound by ZNF143 *in vitro*.

**Figure 6:**
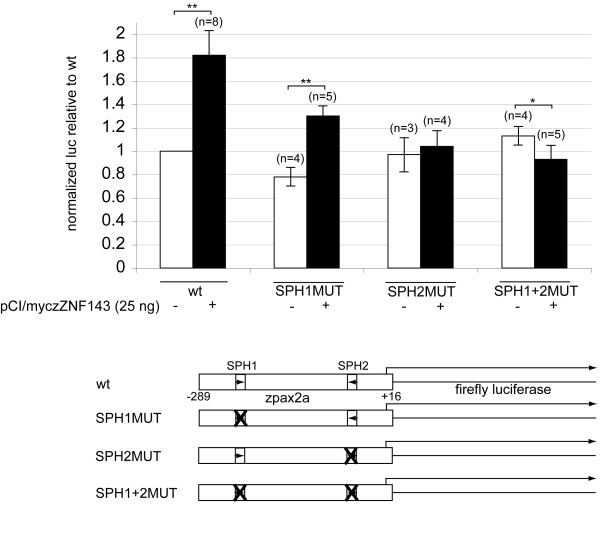
**SPH sites in the zebrafish *pax2a *promoter are functional for transcription in transfected ZF4 cells**. Zebrafish ZF4 cells were transfected per well with 200 ng plasmid DNA containing wt or SPHMUT *pax2a *promoters driving firefly luciferase expression, plus 200 ng pRL-SV40 renilla luciferase plasmid as a normalization control, plus 25 ng of either empty vector pCI-basic plasmid (unfilled columns) or pCI/myczZNF143 to express myc-tagged full-length ZNF143 (filled columns). The ratio of luciferase/renilla light units was determined for each sample, and these ratios were compared relative to the wt promoter with no transfected ZNF143 plasmid included in every experiment. The height of each column represents the average value for the number of independently transfected samples (specified in parentheses), and error bars depict one standard deviation above and below the mean. A double asterisk signifies a p-value <0.01, and a single asterisk signifies a p-value <0.05 comparing like reporter samples with and without addition of ZNF143 expression plasmid.

Next we investigated the transcriptional effect of the SPH sites in the zebrafish *pax2a *promoter in transient transfection experiments. Luciferase reporter plasmids containing either the wt *pax2a *promoter (-289/+16), single SPH1 or SPH2 mutants, or the double SPH1/2 mutant were added to zebrafish ZF4 cells. All mutant sites destroyed the highly conserved CCCA sequence of a SPH element (mutant sequences are shown in Methods). The efficacy of the SPH2MUT alteration was demonstrated by the obliteration of the DNaseI footprint *in vitro *(Figure 5C, lanes 6-9). The zebrafish *pax2a *proximal promoter (-289/+16) was quite active in transfected ZF4 cells since a typical experiment yielded approximately 7-fold higher normalized firefly luciferase level than from a simple TATA box only promoter (G5 reporter in Figure 3A). In transfected ZF4 cells, using a synthetic promoter containing multiple SPH elements, we found only low, basal activity at the same level as from a promoter lacking SPH sites (Figure 3A, compare columns 1 and 2). In human HEK293 cells the SPH5 promoter activity is approximately 20-fold higher than from the G5 promoter (unpublished results). Hence, unlike HEK293 cells, ZF4 cells do not contain sufficient amounts of active ZNF143 to stimulate potentially responsive promoters. Transcription from the wt *pax2a *promoter was increased almost two-fold by co-transfection with a ZNF143 expression plasmid (Figure 6). The SPH2MUT and doubly mutant promoters were not activated, whereas the relative stimulation of transcription from the SPH1MUT promoter was somewhat reduced (Figure 6, filled bars). Not surprisingly, transcription from the various SPHMUT *pax2a *promoters was not reduced significantly in the absence of exogenous ZNF143 (unfilled bars in Figure 6). Hence the SPH1 and SPH2 elements are functional for transcriptional activity directed by the zebrafish *pax2a *gene promoter.

## Discussion

In this study, we show that MO-induced knockdown of ZNF143 results in many phenotypic effects during zebrafish development. General defects in tail formation were obvious, but we also noticed abnormal heart, blood, ear and MHB development. The specificity of the MO-induced phenotypes was confirmed by experiments showing phenotypic rescue by the full-length protein but not a truncated variant lacking transcriptional activation domains. In the morphants, expression of *gata1 *(blood), *cmlc2 *(cardiac) and *pax2a *(MHB) were reduced or altered spatially. Furthermore, we found two binding sites for ZNF143 in the zebrafish *pax2a *gene proximal promoter, and disruption of these sites reduced transcription in transfected zebrafish cells. Therefore, one likely mRNA gene target for transcriptional control by this protein *in vivo *is *pax2a*, although the number is probably much higher.

It is not surprising that a multitude of phenotypes appear after ZNF143 knockdown because many vertebrate small RNA genes contain SPH sites, and in mammals it has been hypothesized that approximately 2000 mRNA gene promoters contain SPH sites [5]. It seems likely that the gene encoding ZNF143 is essential for zebrafish viability. The number of promoters containing SPH elements in zebrafish is unknown. Previously, we have demonstrated an important positive role for the SPH site in zebrafish *U6 *snRNA gene promoters [29], and have noticed putative SPH elements in many zebrafish small RNA gene promoters (unpublished results). Many fugu small RNA gene promoters contain SPH sites [23]. At present, we do not know which phenotypic effects or the relative severity of those effects caused by ZNF143 knockdown are the result of altered expression of small RNA genes or mRNA genes. Since knockdown was rescued by coinjection of synthetic mRNA encoding full-length ZNF143, but not by RNA lacking coding potential for N-terminal activation domains (Figure 3), we hope to be able to distinguish differential effects of mRNA-activation vs. small RNA activation by this protein using rescue with synthetic RNA encoding deletions or mutations in those separable activation functions.

Since knockdown of ZNF143 caused relatively drastic phenotypic effects in zebrafish embryos, we were surprised that cultured zebrafish ZF4 cells contain undetectable levels of endogenous active protein in a transactivation assay (Figure 3A, compare reporter gene expression from G5 promoter vs. SPH5 promoter in columns 1-3). When these same reporter constructs were used in transfected human HEK293 cells the SPH5 promoter was transcribed at approximately 20-fold higher level due to endogenous ZNF143. Perhaps a small amount of ZNF143 in ZF4 cells is sufficient for their growth, and that amount is below a threshold detectable with relatively large amounts of reporter plasmid added in the transfection experiments. Indeed, mRNA encoding this protein in ZF4 cells was detected by RT-PCR (results not shown). Another possibility is that relative overexpression of ZNF143 in transfected cells suppresses another defect in the small RNA activation pathway in ZF4 cells. Nevertheless, the nonsaturating levels of active ZNF143 in ZF4 cells allowed us to use them as an assay for mRNA activation potential of tagged protein and protein lacking activation domains (Figure 3A), and we will use this assay in the future to examine activity of mutant proteins.

Our experiments demonstrating brain defects and, specifically, reduced *pax2a *expression upon ZNF143 knockdown induced us to inspect the *pax2a *proximal promoter for SPH sites. Indeed, ZNF143 binds to the *pax2a *promoter *in vitro *and activates it in an SPH-site dependent manner when cotransfected into ZF4 cells. Although we used existing human ZNF143 protein reagents in our laboratory for the binding studies, rather than the zebrafish protein, we expect that nucleotide specificity of the human and zebrafish proteins should be undistinguishable. The zinc finger DNA-binding domains are 91% identical, with no changes in the amino acid residues known to be most important for DNA recognition by this motif [23,29,30]. Although the enhancer(s) of the zebrafish *pax2a *gene promoter have been characterized partially [31], we are not aware of previous work to investigate elements within the proximal promoter. We expect that other elements in addition to the SPH1 and SPH2 sites constitute this promoter. Luciferase reporter gene activity of the SPH1+2 mutant promoter was approximately 7-fold higher than that found with a simple TATA-containing promoter in our transient transfection assays (unpublished results). This promoter does not contain a readily identifiable TATA box, a characteristic that is true also of most previously identified SPH-containing mammalian mRNA gene promoters [5]. It will be interesting to dissect the *pax2a *promoter further in order to discern relative roles of SPH elements and other elements with respect to both basal transcription and enhancer-driven expression.

Because of the widespread abundance of SPH sites in mammalian mRNA gene promoters, we searched for them in other important developmental regulators in addition to *pax2a*. Although several SPH sites appeared to be candidates in the zebrafish *gata1 *proximal promoter, none bound ZNF143 *in vitro *(unpublished results). On the other hand, we have found a high-affinity SPH element in the zebrafish fibroblast growth factor receptor-1 (*fgfr1*) and glycogen synthase kinase 3α promoters (unpublished results). Whether any of the aforementioned genes is controlled in a significant manner by ZNF143 will require further study. Furthermore, because of the large potential number of target promoters in mammals, it is highly likely that the transcription of many other developmental regulator genes is controlled by ZNF143.

## Conclusions

Because knockdown of the transcriptional activator ZNF143 by injection of translational-blocking MOs causes many significant phenotypic effects in zebrafish embryos, we conclude that this protein is essential for normal development. Phenotypic effects are rescued by coinjection of full-length znf143 mRNA, but not by mRNA lacking coding capacity for the N-terminal region containing activation domains. Hence, the MO-mediated knockdown is specific, and ZNF143 function in vivo requires the N-terminal region. Furthermore, the pax2a gene promoter is at least one likely target of ZNF143 during zebrafish development.

## Methods

### Plasmid constructions

A cDNA containing the zebrafish *znf143 *gene was obtained from Open Biosystems. We named this plasmid pME18S-FL3/zZNF143. The open reading frame (ORF) contained within this insert was lacking a full-length gene and contained a reading frame error in the coding region near the amino-terminus of the encoded protein. Hence, in order to construct a full-length ORF situatated behind a T7 promoter, three DNA fragments were ligated as follows. The "correct" amino-terminal region fragment was prepared by reverse transcriptase polymerase chain reaction (RT-PCR) using total RNA from zebrafish ZF4 cells and ligated into a pGEM-T vector (Promega). This fragment was excised using *Kpn*I and *Pvu*II and purified by agarose gel electrophoresis. The main body of the *znf143 *ORF was excised from pME18S-FL3/zZNF143 using *Pvu*II and *Xho*I and purified by agarose gel electrophoresis. The third DNA fragment was the pBlueScript SK vector (Stratagene) opened at *Kpn*I and *Xho*I sites, and purified by chromatography on Sepharose CL-4B. The three-fragment ligation reaction was used to transform *E. coli *XL1-Blue (Stratagene) competent cells. The resulting plasmid was named pBS/zZNF143. A single myc tag was inserted at the amino-terminus of the encoded ZNF143 protein using the QuickChange site-directed mutagenesis protocol. The subsequent Δ2-225 deletion within the zebrafish *znf143 *ORF started with the parent plasmid pBS/myczZNF143 and was constructed using the QuickChange protocol in which the oligonucleotides base-paired across the deletion endpoints and looped out the template DNA to be deleted. Plasmid minipreps were prepared using a Qiagen miniprep kit, and such preparations were suitable for mRNA synthesis *in vitro*. Zebrafish ZNF143 expression plasmids used for transient transfection experiments were constructed by amplifying inserts from pBS-based plasmids using PfuUltraHF polymerase (Stratagene), restriction with *Xba*I and *Sal*I, gel-purification, and ligation into like sites in the pCI-neo vector (Promega). GAL4DBD - zebrafish ZNF143 fusion expression plasmids were constructed in the pCI-neo vector (Promega), and contained inserts encoding the amino-terminal 94 aa of *S. cerevisiae *Gal4p [11] fused in-frame to zebrafish ZNF143 fragments amplified by PCR. Luciferase reporter plasmids used for transient transfection experiments were based upon the pGL3-basic vector (Promega) and included the adenovirus major late basal promoter (AdMLT). The pGL3/G5AdMLT ("G5") reporter contained five copies of the *S. cerevisiae *upstream activating sequence galactose (UAS_G_) element ligated upstream of the TATA box, whereas the "SPH5" reporter contained five copies of the human *U6-1 *gene SPH element in the same position. The zebrafish *pax2a *gene promoter (-289 to +16) was amplified from genomic DNA by PCR and ligated into the pGEM-T vector (Promega). The location of the transcriptional start site (+1) is according to the 5'-most expressed sequence tag (EST) in the University of California, Santa Cruz zebrafish genome assembly (July 2007 assembly, chromosome 13, position 29,268,562). SPHMUT plasmids containing clustered point mutations within SPH sites of the *pax2a *promoter were prepared by the QuickChange protocol. The nucleotide changes introduced into SPHMUT sites were (mutant nucleotides in lower case letters): SPHMUT1: 5'-TAAgatcTCTCTCCTCAT-3'; SPH2MUT: 5'-CTagatctATCCCCCCTC-3' (bottom strand); SPH3MUT: 5'-CAagatctAGCTTCTAAC-3' (top strand). DNA fragments containing wt and mutant *pax2a *promoters were amplified by PCR using pGEM-based templates, restricted with *Sac*I and *Nhe*I, and ligated into the same sites of the pGL3-basic vector. Inserts of all plasmids were sequenced completely to verify deletions, in-frame ligations, and ensure that no other mutations were added to the *znf143 *ORF or *pax2a *promoter. DNAs used for transient transfection experiments were purified using the Qiagen plasmid maxi kit, and concentrations were determined by absorbance at 260 nm.

### Cell culture, transfection, and reporter gene assays

Human HEK293 cells were purchased from ATCC (CRL-1573) and cultured in Dulbecco's Modified Eagle Medium (DMEM), containing high glucose (Gibco 11995), penicillin-streptomycin, and 10% bovine growth serum (Hyclone). Experiments with HEK293 cells were performed under BSL-2 guidelines following approval by the Texas A&M University Institutional Biosafety Committee. Zebrafish ZF4 cells [32] were obtained from ATCC (CRL-2050) and grown in DMEM/F12 medium (Gibco 11320), penicillin-streptomycin, and 10% bovine growth serum. Cells were transfected in 6-well dishes using Lipofectamine 2000 (Invitrogen) following the manufacturer's protocol with amounts of DNA described in the figure legends. Cells were co-transfected with pRL-SV40 (Promega), a renilla luciferase reporter, for normalization. 48 h post-transfection, cell extracts were prepared, and firefly and renilla luciferase activities were measured with a Sirius tube luminometer (Berthold) using reagents and protocols from the Dual-Luciferase Reporter Assay System (Promega). The number of independently transfected samples for each condition is shown in parentheses above the bars in the graphs. Statistical significance (p-values) comparing sets of different experiments was calculated using the Student's T test in Microsoft Excel.

### Electrophoretic mobility shift assay (EMSA) and DNaseI footprinting

Two radiolabeled *pax2a *gene proximal promoter probes used for protein-DNA binding assays were prepared by PCR using either ^32^P-end-labeled CZPAX2A-131 primer (5'-CAACACTTTGTGATTCGCCAACGC-3'), and unlabeled ZPAX2A-289 primer (5'-TGGTACCGCTTCCTTTCCACTTGT-3'), or ^32^P end-labeled CZPAX2A+16 primer (5'-ATGTGCCTGTTAGAAGCTTTGGGC-3') and unlabeled ZPAX2A-154 primer (5'-GCGTTGGCGAATCACAAAGTGTTG-3'), and pGEM plasmid DNA with wt or SPHMUT *pax2a *promoter inserts. The zebrafish *U6-1 *probe was prepared as described [29]. Protein for EMSA experiments was prepared by *in vitro *transcription/translation of human *znf143 *as described previously [9]. DNA-protein complex formation, competition with oligonucleotides, and electrophoresis on non-denaturing gels were performed as before [33]. Sequences of the competitor oligonucleotides can be found in [2] where human *U6-1 *SPH is called NONOCT(long) and human *U6-1 *OCT is called OCTCON. DNaseI footprinting assays followed the same protocol as described previously [33], using purified human ZNF143 zinc finger DNA-binding domain (DBD) (amino acids 236-444) expressed from a pET5a vector in *E. coli *[29]. The positions of the putative SPH elements were deduced by electrophoresis of T and C dideoxy sequencing reactions on the same gels.

### Zebrafish husbandry and embryo microinjection

AB strain zebrafish were maintained using standard methods [34], and with protocols approved by the Texas A&M Animal Care and Use Committee (AUP #2007-90 and 2010-90). MOs and synthetic mRNAs were injected at the one- to two-cell stage. Two MOs were designed to block the translation of zebrafish *znf143*, dissolved in 1X Danieau buffer, and were coinjected at a concentration of 2 ng/nL, each. The "Start" MO, ordered from Open Biosystems and Gene Tools, LLC, had the sequence, 5'-ATTCACCTGGGCTAACAGCATGATC-3', and the "5'UTR" MO, ordered from Gene Tools, LLC, had the sequence, 5'-CAACAATCCCTTCGTTCGACCACCA-3'. The control MO (5'-CCTCTTACCTCAGTTACAATTTATA-3') was purchased from Gene Tools, LLC. For rescue coinjection experiments, capped and polyadenylated mRNAs were synthesized from pBS/myczZNF143 or the Δ2-225 deletion variant template using the mMESSAGE mMACHINE T7 Ultra kit (Ambion). Synthetic mRNA concentrations were determined using a NanoDrop spectrophotometer. Approximately 3 nL of RNAs were injected at a concentration of 30 pg/nL, an amount that did not cause any toxicity or phenotype when injected without MOs. Phenotypes were classified for quantitation in Figure 3C at 48 hpf.

### Immunoblots

Nuclear extracts from HEK293 cells were prepared using the NE-PER kit (Pierce/Thermo Scientific). After electrophoresis on standard SDS denaturing gels, proteins were transferred to nitrocellulose, and probed with rabbit anti-yeast GAL4DBD (Upstate/Millipore), or mouse monoclonal anti-myc (Upstate/Millipore, clone 4A6).

### Whole-mount in situ hybridization

Whole-mount in situ hybridizations with embryos were performed according to standard methods [26]. Probes used were *cmlc2 *[35], *pax2a *[36], *gata1 *[28], *myoD *[27], *gsc *[24], *otx2 *[25], and *egr2a *(or *krox20*) [26]. The numbers of independently injected embryos scored for various phenotypes are noted in the text.

## Authors' contributions

KMH designed and carried out experiments shown in Figures 2, 3C, and 4. ACL participated in the design of the study, the microscopy and the preparation of the manuscript. GRK performed experiments shown in Figures 1, 2A,B, 5, and 6, conceived the project, coordinated the work, and drafted the manuscript. All authors read and approved the final manuscript.
